# Decision support system for ranking relevant indicators for reopening strategies following COVID-19 lockdowns

**DOI:** 10.1007/s11135-021-01129-3

**Published:** 2021-04-10

**Authors:** Tarifa S. Almulhim, Igor Barahona

**Affiliations:** 1 Department of Quantitative Methods, School of Business, King Faisal University, P.O.Box 400, Al-Ahsa, 31982 Saudi Arabia; 2 Laboratory of Applications of Mathematics, Institute of Mathematics, Universidad Nacional Autónoma de México (UNAM), 62243 Cuernavaca City, Morelos México

**Keywords:** COVID-19, Lockdowns, Decision support system, Interval valued intuitionistic fuzzy sets, Analytic hierarchy process

## Abstract

The pandemic caused by the spread of the SARS-CoV-2 virus forced governments around the world to impose lockdowns, which mostly involved restricting non-essential activities. Once the rate of infection is manageable, governments must implement strategies that reverse the negative effects of the lockdowns. A decision support system based on fuzzy theory and multi-criteria decision analysis principles is proposed to investigate the importance of a set of key indicators for post-COVID-19 reopening strategies. This system yields more reliable results because it considers the hesitation and experience of decision makers. By including 16 indicators that are utilized by international organizations for comparing, ranking, or investigating countries, our results suggest that governments and policy makers should focus their efforts on reducing violence, crime and unemployment. The provided methodology illustrates the suitability of decision science tools for tackling complex and unstructured problems, such as the COVID-19 pandemic. Governments, policy makers and stakeholders might find in this work scientific-based guidelines that facilitate complex decision-making processes.

## Introduction

Virology scientists believe that there are approximately 1.7 million undiscovered types of viruses that are hosted only by mammals and birds. These scientists have no precise information about their nature, how they are transmitted to humans and spread, or their accompanying symptoms (Carroll et al. 2018). Therefore, the probability of humans becoming infected with a previously unknown virus that could lead to a new pandemic is unknown. In December 2019, the world witnessed the emergence of a new virus that was found in the city of Wuhan, China. Since that time, the spread of coronavirus disease 2019 (COVID-19), which is caused by severe acute respiratory syndrome coronavirus 2 (SARS-CoV-2), has increased exponentially. According to the John Hopkins University of Medicine Coronavirus Resource Center website, by the end of January 2021, there were approximately 108, 148,755 infected people and 2,267,768 deaths across 188 countries (JHU 2021). The common symptoms reported by infected persons include fever, shortness of breath and several respiratory disorders. Saliva droplets and physical contact are reported as the main modes of transmission. As a result of limitations in terms of epidemiological surveillance and partly, diagnostic capacity, confirmed cases are likely to be underestimated in most countries (Jayaweera et al. 2020).

Considering that the virus spreads very rapidly, it was deduced that at some point, healthcare systems around the world would collapse. COVID-19 forced governments to implement mitigation strategies from restricting non-essential activities, for example, by closing schools, shopping centres and amusement parks, to instituting severe curfews with strict travel and movement restrictions. While most lockdown protocols implemented by countries considered the need to maintain access to important services, differences in the extent to which other non-essential activities were closed or opened were found across countries. For instance, the Saudi Arabian government implemented a nationwide full curfew from April 6 until May 28 that included 24 h movement restrictions all over the country (Anil and Alagha 2020). Mexico implemented a mitigation strategy that comprised lockdowns that restricted all non-essential activities. Although the government encouraged the populace to voluntarily reduce its mobility and carry out only essential activities, compulsory restrictions were not imposed (Estévez-Soto 2020). Unlike the previously mentioned countries, Sweden did not impose strict lockdowns, and an important part of its economic sectors remained opened (Carbone and Montecucco 2020). It is clear that different levels of lockdowns yield different results. In this regard, there is plenty of literature that contrasts performance and outputs among different types of lockdowns, strategies and scenarios across countries (Dickens et al. 2020; Sardar et al. 2020; Shammi et al. 2020). Months after the emergence of SARS-CoV-2, the debate has moved to identify the best strategy for returning “new regularity” by minimizing risk and maximizing benefits. In this context, the main objective of this work is to propose a decision support system (DSS) that aids governments, policymakers, or decision makers for assessing the most relevant indicators while a reopening strategy is designed after COVID-19 lockdowns. By identifying a set of indicators that should be prioritized after Covid-19 lockdowns through replicable and evidence-based methodology, we make a significant contribution for achieving a deeper understanding of this pandemic. The main “take-away” from this work available to readers, practitioners and stakeholders are given by the flexibility and capability of our methodology, which can be adapted to different contexts and scenarios. The investigated indicators are grouped into four dimensions: economic growth, environmental preservation, well-being in society and individual health. Note that these indicators are utilized by worldwide organizations as International Labour Organization (ILO 2021), International Monetary Fund (IMF 2021), Food and Agricultural Organization (FAO 2021) for investigating, ranking, or comparing countries. In this form, indicators proposed by mentioned organizations represent a framework which facilitates the replicability of this methodology across countries.

The paper is structured on six sections. A literature review, which comprises definitions of indicator criteria and a revision of the existing work in the field, is provided in the next section. The proposed methodology is introduced in section three. The results are provided on section fourth. A discussion that contrasts our results with another research is presented in the fifth section. The last section presents the conclusions and future research directions.

## Contextual framework (Problem description)

There is scientific evidence that lockdowns and curfews, which comprise strategies such as social distancing, working at home and school closures, are extremely useful to “flatten the curve” of the number of infected people and therefore play an important role in avoiding overdemand for health care services in a short period of time. Along 2020, mathematical models and data science projects proliferated around the world as support systems for local governments to make better informed decisions about lockdown objectives and scopes (Ibarra-Vega 2020; Sardar et al. 2020). When forecasting how a pandemic is evolving, the general approach in epidemiology is centred on three variables: infected, deaths and recovered, which are also are non-linear functions with parameters given by the mitigation policies. Comorbidities and sociodemographic factors can be included in the model as covariates (Medrek and Pastuszak 2020). As long as the number of infected people is lower than the healthcare capacity, the pandemic remains manageable. Considering this the main objective of lockdowns and curfews, parallel debates emerged in relation to the collateral effects from another perspectives: economic, environmental, public and individual health (Martin et al. 2020). Quarantines and lockdowns reduce pandemic intensity but make it last longer. Regarding collateral effects, studies have estimated that lockdowns shorten economic activity by at least 40% (Martin et al. 2020). The International Monetary Fund (IMF) expected that the global economy will contract by 4.7% by 2020 (IMF 2021). The International Labour Organization reported that unemployment reached its peak point by reaching levels of approximately 6.7% of the global force, which is approximately equal to 195 million full-time employees (ILO 2021). Similarly, COVID-19 lockdowns, mainly related to confinement and isolation, brought several mental health problems, such as stress, depression and suicide. Because COVID-19 is an emerging phenomenon, studies accurately reporting repercussions on individuals’ mental health are not fully available. In 2003, SARS-CoV-1 was associated with a 30% increase in suicide in those aged 65 and older. Approximately 50% of the influenza-recovered patients were diagnosed as stressed and anxious, and approximately 30% of the healthcare workforce at health services showed emotional distress (Lee et al. 2007). However, not all side effects of COVID-19 are negative or undesirable. Recent data released by NASA (National Aeronautics and Space Administration) and the ESA (European Space Agency) indicated that as a result of quarantines and mobility restrictions, air pollution in Wuhan, Italy and Spain was reduced by approximately 30% (Cicala et al. 2020). Another study demonstrated that CO2 levels showed important reductions (30.3–48.5%) in the metropolitan area of Rio de Janeiro Brazil as a result of the COVID-19 quarantine (Dantas et al. 2020).

While analysing the impact of lockdowns on human activities, it is important to follow a systematic approach which considers all outputs, either positive, negative or neutral. There is supportive evidence that systematic approaches yield to better-informed decision-making processes and more accurate results (Pileggi 2020). In this case, it is evident that outputs related to COVID-19 lockdowns might conflict or be negatively correlated. While the main purpose of quarantines is to minimize the number of deaths caused by the virus, this is achieved by incurring collateral costs. When the time for reopening economies and societies has arrived, decision makers (DMs) must select the most suitable indicators by analysing the negatively correlated variables or conflicting criteria. Consider the following illustrative example: let “A” and “B” represent the relevant indicators to be assessed to determine whether a lockdown should be lifted. From one perspective, “A” can be considered better than “B”, and from a different point of view, “A” is better than “B”. Conflictive scenarios occur when indicators can be grouped simultaneously in two or more opposite categories, named “benefits” or “costs”. In this context, multi-criteria decision-making (MCDM) is suitable for decision-making problems with conflicting, incomplete or contradictory information. Research that proposes different types of Decision Support Systems based on MCGDM methods in the context of COVID pandemic recently emerged. Ashraf and Abdullah (2020) propose a decision-making approach for investigating COVID pandemic under the spherical fuzzy environment. Their results include indicators for measuring the degree of global emergency and uncertainty due to the pandemic. An application of DEMATEL method with intuitionistic fuzzy sets for modelling relaxations in lockdowns, on Ocampo and Yamagishi (2020) is proposed. By the application of TOPIS, Shrestha et al (2021) investigate the consequences of COVID pandemic on globalization. Due to space limitations other works related to this field were not mentioned. Readers interested on more details are advised to get Garg and Kumar (2019).

Most MCDM problems require the participation of more than one DM in the process. Hence, many MCDM approaches are employed in a group decision-making (GDM) environment. Since the emergence of multi-criteria GDM (MCGDM) methods in the early 1970s, they have been applied in different disciplines and contexts. MCGDM methods have been demonstrated to be suitable for finding solutions to complicated problems with conflicting criteria (Park et al. 2008; Deshpande et al 2020; Rowley et al. 2020; Pileggi, 2020). During the past decade, methodologies such as the analytic hierarchy process (AHP), technique for order of preference by similarity to ideal solution (TOPSIS), evidential reasoning (ER) and Višekriterijumsko kompromisno rangiranje (VIKOR), among others, have been widely applied in different disciplines and contexts (Yang et al. 1994; Hsu and Hsu 2008; Park et al. 2008; Zhao et al. 2013; Büyüközkan and Göçer 2018; Toros and Gazibey 2018; Lei et al. 2020). AHP is a mathematical method that places decision objects into a simple and practical hierarchical structure (Saaty 1977). The hierarchical shape, which is characteristic of AHP, allows the decision objects to align with the overall goal. The hierarchy simplifies the comprehension of problems with multiple aspects that are often affected by complex relations and therefore enables DMs to assess relationships in a structured and systematic way (Abdullah and Najib 2016). By using crisp values, pairwise comparisons are performed, and ultimately, overall weights for each criterion are obtained. The first versions of AHP were based on crisp numbers, and later, its limitations for accurately describing real-world problems were noted (Abdullah and Najib 2016; Büyüközkan and Göçer 2018). Because real-world problems are mainly characterized by vagueness and uncertainty, intuitionistic fuzzy set (IFS) theory was incorporated into AHP. Then it was extended to the fuzzy environment by incorporating concepts from interval-value intuitionistic fuzzy (IVIF) set theory (Atanassov 1986). This method is popularly known as intuitionistic fuzzy AHP (IVIF-AHP) (Abdullah and Najib 2016). By differentiating membership, non-membership and hesitancy, the IVIF-AHP has proven to be a reliable method for representing DM preferences as intuitionistic fuzzy values rather than exact numbers (Merigó and Casanovas 2011).

According to Abdullah and Najib (2016), IVIF-AHP can use incomplete information to accurately standardize scores across DMs. To overcome this limitation, the fuzzy cross entropy of IVIF sets was introduced (Ye 2011). By considering the optimal weights of the indicators, DM preferences can be aggregated, and the product of each pair of interval-value intuitionistic fuzzy number (IVIFN) matrices can be obtained. Considering this framework, several authors proposed the utilization of IVIF-AHP, where the IVIF numbers were obtained from linguistic variables rather than natural numbers (Wang et al. 2015; Abdullah and Najib 2016). As illustrated in the next section, IVIF-AHP can be employed to formalize linguistic variables and therefore provides a more transparent decision-making process. Taking in to account the characteristics of the problem, DMs might either come from different areas of expertise or not. The entropy weight method is the most common method and is capable of determining the weights of the DMs in decision-making processes (Ye 2011).

Under this framework, it is relevant for policy makers, governments and DMs to utilize a DSS that assigns importance to indicators to design a reopening strategy once COVID-19 lockdowns have been lifted. The present work introduces a novel interval-value intuitionistic fuzzy sets-DSS (IVIFS-DSS) methodology based on MCGDM approaches to identify the most important indicators that should be considered when a reopening strategy is implemented. A total of 16 criteria, which are grouped into four “dimensions”, are evaluated with respect to their impact on a given reopening strategy (see Table 1).

**Table 1:** Definitions of the key variables included in this research

Dimension and its indicators/criteria	Brief definition	Related literature
*Economy (C*_*1*_*)*		
Gross domestic product (C_11_)	The monetary value of all final goods and services that are bought by final users and produced in a given country or region over a certain period of time	OECD (2021b), IMF (2021)
Inflation (C_12_)	A continued increase in the general level of prices over a period	Işığıçok et al. (2020)
Retail sales (C_13_)	All purchases of finished goods and services by final consumers or businesses	Amadeo (2020)
Industrial production (C_14_)	All outputs of the industrial sector over a specific period of time. It typically includes the production of three main industries: manufacturing, mining and utilities	Shapiro et al. (1989), Hudson (2020)
*Environmental protection (C*_*2*_*)*		
Carbon dioxide emissions (C21)	The release of CO2 to the atmosphere is defined greenhouse gas emissions, which are among the main causes of global warming	US—EIA (2020a), Eurostat (2020)
Solid waste (C22)	Garbage including refuse; mud from wastewater treatment plants, water supply treatment plants, and air pollution control stations; and other castoff material	US—EIA (2020b), US—EPA (2020)
Freshwater source preservation (C23)	Any naturally existing water, except water flowing from seas and oceans, is defined as fresh water. The preservation of fresh water is vital for human life	Beatley (2000)
Forest preservation (C24)	A forest is a land that comprises more than 0.5 hectares with trees higher than 5 m and a canopy cover of more than 10%	FAO (2021a), Collins et al. (2012)
*Societal well-being (C*_*3*_*)*		
Poverty reduction (C31)	Poverty refers to the condition in which individuals lack the financial resources to achieve a minimum standard of living	Barahona (2018)
Reduction in unemployment (C32)	Generally, unemployment is defined as the state of being without work while looking for employment	ILO (2021)
Zero CO2 emissions mobility (C33)	Transport activities represent the main source of CO2 emissions. Lockdowns reduced travel transportation by approximately 40%. Since clean air is vital for humans, this indicator is important	US–CB (2020)
Reduction in crime and violence (C34)	Violence is the use of physical force to injure, abuse, damage, or destroy. Strategies to mitigate crime and violence should be considered when COVID lockdowns are lifted	WHO (2021), Aboal et al. (2016)
*Individual well-being (C*_*4*_*)*		
Mental health (C41)	Mental health refers to our emotional and psychological well-being and is related to an individual’s capability to handle stress, interact with others and make decisions	US–DHHS (2020)
Physical wellness (C42)	Physical wellness consists of engaging in regular physical movement, eating a nutritious diet, proper sleeping and engaging in safe behaviours	Wu and McGoogan (2020), Brooks et al. (2020)
Education (C43)	Education refers to the process of facilitating learning or the acquisition of knowledge, skills, values, beliefs, and habits	Kjällander et al. (2018)
Nutrition (C44)	Nutrition refers to the assimilation of food and other nourishing materials by the body. Individuals with access to balanced diets can strengthen their immunity system and therefore are less like to become sick	Jaggers et al. (2020)

The reason for selecting these indicators and no others is based on a literature review carried out for these purposes. According to worldwide organizations such as the United Nations (UN), Organization for Economic Cooperation and Development (OECD), Bank for International Settlements (BIS) and Food and Agriculture Organization of the United Nations (FAO), among others, the cited indicators are widely utilized for ranking, comparing or investigating countries (FAO 2021b; OECD 2021a; OECD 2021b; UN 2021). Note that some of the mentioned organizations gather more than 100 countries. These indicators represent a solid framework which allows the replicability of our methodology across countries. It is beyond the scope of this word to provide a detailed operational definition of these indicators; however, summarized definitions are provided for these indicators in “Appendix 1”.

Our work aims to assist DMs and policy makers by identifying the best mix of indicators that should be considered when a reopening strategy is implemented. The novel contributions of this research are as follows:

- This study identifies 16 indicators in four categories, the economy, environmental protection, societal well-being and individual health and ranks their importance for reopening strategies after COVID-19 lockdowns have been lifted.
- This study employs a novel IVIFS-DSS methodology based on MCGDM approaches (entropy weighting and AHP) to help policy makers prioritize and select indicators that must be considered when a reopening strategy is carried out.
- This study provides practical guidance that can help policymakers and planners make better decisions during the ongoing pandemic and future pandemics.

## Materials and methods

This section gathers the theoretical foundations behind the proposed methodology. It is composed by three subsections. Formal definitions for IFS and IVIFS are firstly presented. They are followed by detailed explanations of linguistic variables and theirs respective IVIFN equivalences. At the end, the IVIF DSS methodology, which comprises eight steps, is presented. For sake of simplicity, note that section four follows the same eight-step sequence that is here presented at subsection three.

### Definition of IVIFS

After ordinary fuzzy set theory was proposed by Zadeh (1965) to address the uncertainty of human judgement, an improved extension referred to as the IFS was introduced by Atanassov (1986). If $\stackrel{\sim }{X}$ denotes an IFS bounded in domain *Z*, then it can be expressed in the following way.

1 $$\widetilde{ X} = \left\{ {\left\langle {z,\mu_{{\widetilde{{\tilde{X}}}}} \left( z \right), \nu_{{\tilde{X}}} \left( z \right)} \right\rangle /z \in Z} \right\}$$

**Fig. 1:**
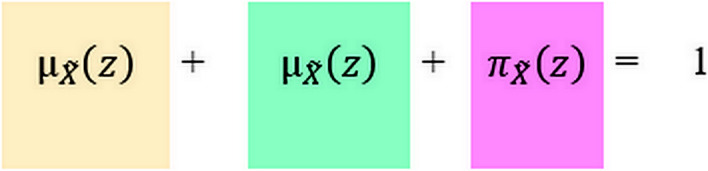
Membership, non-membership, and hesitancy relations

In formula (1), ${\mu }_{\stackrel{\sim }{X}}\left(z\right)$ and ${\nu }_{\stackrel{\sim }{X}}\left(z\right)$ represent the membership degree and non-membership degree, respectively. Furthermore, $z\in Z$ for any subset $\stackrel{\sim }{X}$ of $Z$, and ${\mu }_{\stackrel{\sim }{X}}\left(z\right)$ and ${\nu }_{\stackrel{\sim }{X}}\left(z\right)$ satisfy $0\le {\mu }_{\stackrel{\sim }{X}}\left(z\right)+{\nu }_{\stackrel{\sim }{X}}\left(z\right)\le 1$. ${\pi }_{\stackrel{\sim }{X}}\left(z\right)$ represents the hesitancy degree, and therefore, ${\pi }_{\stackrel{\sim }{X}}\left(z\right)=1-{\mu }_{\stackrel{\sim }{X}}\left(z\right)-{\nu }_{\stackrel{\sim }{X}}\left(z\right)$. In this formula, any element $z\in Z$ is assigned to three different categories, as illustrated in Fig. 1.

In some cases, it is not easy to exactly define the membership degrees for certain elements of the IFSs. Thus, Atanassov and Gargov (1989) proposed a generalized type of IFS, which is called IVIFS in the literature. Let *A* be an IVIFS, where $z\in Z$; then, it can be calculated as follows:

2 $$A = \left\{ {z,\mu_{A} \left( z \right), \nu_{A} \left( z \right)/z \in Z} \right\}$$

where ${\mu }_{A}\left(z\right)$ and ${\nu }_{A}\left(z\right)$ take values between [0,1], under the condition that $0\le \mathrm{sup}{\mu }_{A}\left(z\right)+\mathrm{sup}{\nu }_{A}\left(z\right)\le 1$ for any $z \epsilon Z$. The closed intervals of ${\mu }_{A}\left(z\right)$ and ${\nu }_{A}\left(z\right)$ are denoted by the lower and upper bounds as ${\mu }_{A}^{L}\left(z\right)$, ${\mu }_{A}^{U}\left(z\right),{\nu }_{A}^{L}\left(z\right)$, and ${\nu }_{A}^{U}\left(z\right),$, respectively. Note that the intervals of ${\mu }_{A}\left(z\right)$ and ${\nu }_{A}\left(z\right)$ denote the degree of membership and non-membership, respectively, for each $z \epsilon Z$ in the IVIFS $A$, as shown below:

3 $$A = \left\{ {z,\left[ {\mu_{A}^{L} \left( z \right), \mu_{A}^{U} \left( z \right)} \right], \left[ {\nu_{A}^{L} \left( z \right), \nu_{A}^{U} \left( z \right)} \right]/z \in Z} \right\}$$

where ${0\le \mu }_{A}^{L}\left(z\right)\le {\mu }_{A}^{U}\left(z\right)\le 1$ and ${0\le \nu }_{A}^{L}\left(z\right)\le {\nu }_{A}^{U}\left(z\right)\le 1$. Moreover, for each IVIFS $A$, where $z\in Z$, there exists an IVIF index ${\pi }_{A}\left(z\right)$ that denotes the range of the hesitancy membership degree of the element $z \epsilon Z$ in $A$, as proposed by Park et al. (2008) and can be calculated as follows.

4 $$\pi_{A} \left( z \right) = \left[ {\pi_{A}^{L} \left( z \right), \pi_{A}^{U} \left( z \right)} \right] = \left[ {1 - \mu_{A}^{U} \left( z \right) - \nu_{A}^{U} \left( z \right),1 - \mu_{A}^{L} \left( z \right) - \nu_{A}^{L} \left( z \right)} \right]$$

For convenience, an IVIFN is denoted by α. Its formal definition was proposed by Xu and Yager (2006) as $\alpha =\left(\left[{\mu }_{\alpha }^{L}, {\mu }_{\alpha }^{U}\right], \left[{\nu }_{\alpha }^{L}, {\nu }_{\alpha }^{U}\right], \left[{\pi }_{\alpha }^{L}, {\pi }_{\alpha }^{U}\right]\right)$. It is beyond the scope of this work to provide a detailed explanation of the rules, laws and operations of IVIFN. Interested readers are encouraged to consult Park et al. (2008), Büyüközkan and Göçer (2018), Garg and Kumar (2019)

### Linguistic variables

One advantage of IVIFS with respect to other techniques is the possibility of elaborating reasoning assessments, which are given in terms of linguistic variables (e.g., “very good”, “good” and “medium good”) (Wang et al. 2015). Numerous studies have documented that linguistic variables are more suitable for describing real-world MCDM problems that involve vague, imprecise or uncertain information (Garg and Kumar 2019). The linguistic terms used in this work and their corresponding IVIFNs are provided in Table 2. Linguistic variables are efficient and suitable tools for denoting different activities in the real-world that can be assessed by the qualitative judgements of DMs rather than quantitative judgements (Zadeh 1975; Herrera et al. 1997). Linguistic variables are more appropriate for describing real-world MCDM problems involving imprecise and uncertain information (Garg and Kumar 2019). These linguistic variables can be expressed by IVIFNs. Many scholars have concluded that the process of collecting DMs’ judgements for MCGDM problems with a high degree of uncertainty via linguistic contexts leads to more reliable results (Ben-Arieh and Chen 2006; Rodríguez et al. 2013).

**Table 2:** Linguistic terms used to evaluate the importance of the DMs

Linguistic terms	IVIFNs
Extremely knowledgeable (EK)	$([0.95, 1.00], [0.00, 0.00], [0.00, 0.05])$
Very knowledgeable (VK)	$([0.80, 0.85], [0.05, 0.10], [0.05, 0.15])$
Moderately knowledgeable (MK)	$([0.60, 0.65], [0.10, 0.15], [0.20, 0.30])$
Slightly knowledgeable (SK)	$([0.30, 0.35], [0.25, 0.30], [0.35, 0.45])$
Much less knowledgeable (VLK)	$([0.20, 0.25], [0.30, 0.35], [0.40, 0.50])$
Extremely less knowledgeable (ELK)	$([0.00, 0.05], [0.45, 0.50], [0.45, 0.55])$

Note that the IVIFS introduced in Table 2 are utilized here to assess the importance of each DM. The values were proposed by Büyüközkan and Göçer (2018). The linguistic terms’ IVIFNs and reciprocal IVIFNs are presented in Table 3. The relations shown in Table 3 are useful for calculating the relative importance and associated weight for each dimension and its indicators. The methodology proposed by Abdullah and Najib (2016) is replicated here.

**Table 3:** Linguistic terms used for the importance weighting

Linguistic terms	IVIFNs	Reciprocal IVIFNs
Equally important (EI)	$([0.38, 0.42], [0.22, 0.58], [0, 0.4])$	$([0.22, 0.58], [0.38, 0.42], [0, 0.4])$
Intermediate value (IV)	$([0.29, 0.41], [0.12, 0.58], [0.01, 0.59])$	$([0.12, 0.58], [0.29, 0.41], [0.01, 0.59])$
Moderately more important (MMI)	$([0.10, 0.43], [0.03, 0.57], [0, 0.87])$	$([0.03, 0.57], [0.10, 0.43], [0, 0.87])$
Intermediate value (IV2)	$([0.03, 0.47], [0.03, 0.53], [0, 0.94])$	$([\mathrm{0.03,0.53}], [0.03, 0.47], [0, 0.94])$
Strongly more important (SMI)	$([0.13, 0.53], [0.07, 0.47], [0, 0.8])$	$([0.07, 0.47], [0.13, 0.53], [0, 0.8])$
Intermediate value (IV3)	$([0.32, 0.62], [0.08, 0.38], [0, 0.6])$	$([0.08, 0.38], [0.32, 0.62], [0, 0.6])$
Very strongly more important (VSMI)	$([0.52, 0.72], [0.08, 0.28], [0, 0.4])$	$([0.08, 0.28], [0.52, 0.72], [0, 0.4])$
Intermediate value (IV4)	$([0.75, 0.85], [0.05, 0.15], [0, 0.2])$	$([0.05, 0.15], [0.75, 0.85], [0, 0.2])$
Extremely more important (EMI)	$([1, 1], [0, 0], [0, 0])$	$([0, 0], [1, 1], [0, 0])$

Based on the linguistic preferences in the IVIFN and the reciprocal information presented in Table 3, the proposed IVIF-AHP is shaped to prioritize the dimensions and their indicators based on their importance for COVID post-lockdown strategies. Next, the DSS methodology is introduced.

### IVIF DSS methodology

**Fig. 2:**
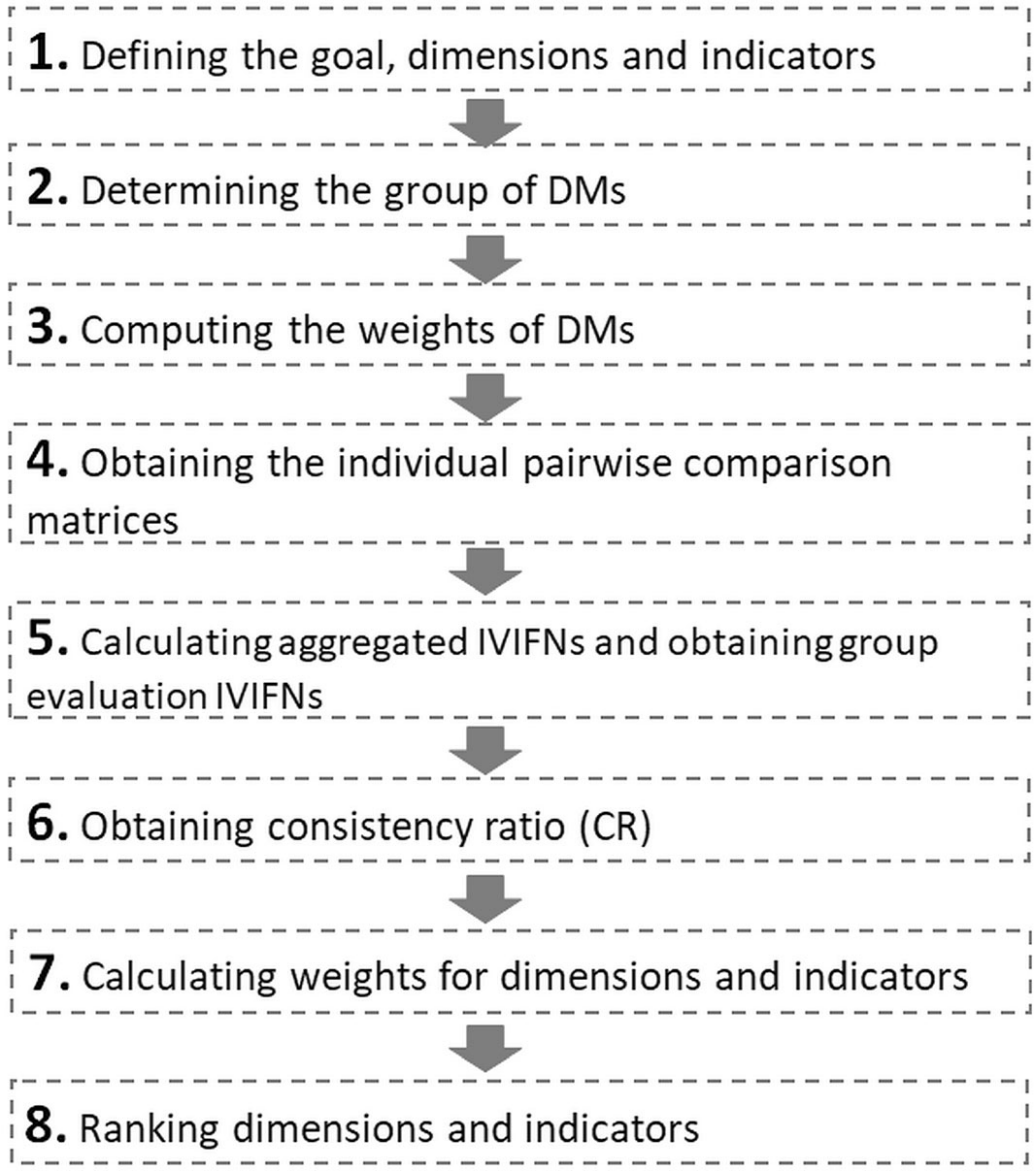
Flowchart of the methodology

Here, we introduce the DSS methodology, which includes eight steps and is based on the IVIF-AHP framework, as illustrated in Fig. 2. These steps are similar to those used for the traditional AHP. However, among the most important differences are the utilization of linguistic variables for collecting DM information and the application of an interval-value measurement scale. The concept of entropy is utilized for assigning weights to each DM according to their respective backgrounds and expertise. The scale is revised by adding interval-value hesitation degrees. These concepts are explained in detail below.

#### Defining the goal, dimensions and indicators

The dimensions and related indicators are defined based on the operational definitions of the variables. Note that the set of indicators aligns with the main objective of the research. A general definition of the problem is provided below:

- Let ${C}_{i}$ be a finite set of $n$ dimensions with a weight vector$[{W}_{1}, {W}_{2}, . . . ,{W}_{n}]$, where ${W}_{i}>0, i=\mathrm{1,2},\dots n, {\sum }_{i=1}^{n}{W}_{i}=1$
- For each $i$-th dimension, let ${C}_{ij}$ be a finite set of $m$ indicators associated with a local weight vector$[{w}_{1}, {w}_{2}, . . . ,{w}_{m}]$, where${w}_{j}>0, j=\mathrm{1,2},\dots m, {\sum }_{j=1}^{m}{w}_{j}=1$. Here, ${C}_{ij}$ denotes the $j$ th indicator of the main $i$ th dimension.
- Let $E = \{{e}_{1}, {e}_{2}, . . . , {e}_{K}\}$ be the same set of $k$ DMs and $E$ be associated with a weight vector denoted as$[{\lambda }_{1}, {\lambda }_{2}, . . . ,{\lambda }_{k}]$, where${\lambda }_{k}>0, k=\mathrm{1,2},\dots K, {\sum }_{k=1}^{K}{\lambda }_{k}=1$.

#### Determining the group of DMs

A GDM is defined as two or more individuals interacting to choose from a set of alternatives. Decisions made by groups are believed to be more robust since they are not attributable to a single individual but rather to the group (Merigó and Casanovas 2011). For real-life situations and problems, evidence shows that collaborative decision-making is one of the most important sources of creativity and accuracy (Ye 2011). Evidence also shows that groups with more than 6 individuals complicate the process (Abdullah and Najib 2016). Therefore, five experts participated in this study.

#### Computing the weights of the DMs

To develop a measure of the importance of each DM in the group in terms of linguistic expressions, three main criteria were considered. (1) Experience refers to the number of years that each DM has been in the professional field since the date of his/her first job. (2) Knowledge refers to the area in which the DM is specialized, and (3) responsibilities indicates whether the DM supervises other people or staff.

${e}_{k} (k=\mathrm{1,2},\dots K)$ represents the participants. Then,$d^{\left( k \right)} = \left( {\left[ {\left( {\mu_{d}^{L} } \right)^{\left( k \right)} , \left( {\mu_{d}^{U} } \right)^{\left( k \right)} } \right], \left[ {\left( {\nu_{d}^{L} } \right)^{\left( k \right)} , \left( {\nu_{d}^{U} } \right)^{\left( k \right)} } \right], \left[ {\left( {\pi_{d}^{L} } \right)^{\left( k \right)} , \left( {\pi_{d}^{U} } \right)^{\left( k \right)} } \right]} \right)$. This measure also includes the uncertainty preference rate for the importance option provided by each DM. Note that the equivalences provided in Table 2 are applied here to obtain numerical values for each${d}^{\left(k\right)}$, where *k* = 1,…,*K* represents the number of DMs participating in the study. Next, a fuzzy entropy measure is applied for each IVIFN to estimate the DM weight vector (denoted as$[{\lambda }_{1}, {\lambda }_{2}, . . . ,{\lambda }_{K}]$) (Ye 2010). Hence, the fuzzy entropy measure of${d}^{\left(k\right)}$, denoted as$EN\left({d}^{\left(k\right)}\right)$, is calculated as follows:

5 $$EN\left( {d^{\left( k \right)} } \right) = \left[ {\cos \left( {\frac{{\pi \left[ {1 + \left( {\mu_{d}^{L} } \right)^{\left( k \right)} + p\left( {\left( {\mu_{d}^{U} } \right)^{\left( k \right)} - \left( {\mu_{d}^{L} } \right)^{\left( k \right)} } \right) - \left( {\nu_{d}^{L} } \right)^{\left( k \right)} - q\left( {\left( {\nu_{d}^{U} } \right)^{\left( k \right)} - \left( {\nu_{d}^{L} } \right)^{\left( k \right)} } \right)} \right]}}{4}} \right) + \cos \left( {\frac{{\pi \left[ {1 - \left( {\mu_{d}^{L} } \right)^{\left( k \right)} - p\left( {\left( {\mu_{d}^{U} } \right)^{\left( k \right)} - \left( {\mu_{d}^{L} } \right)^{\left( k \right)} } \right) + \left( {\nu_{d}^{L} } \right)^{\left( k \right)} + q\left( {\left( {\nu_{d}^{U} } \right)^{\left( k \right)} - \left( {\nu_{d}^{L} } \right)^{\left( k \right)} } \right)} \right]}}{4}} \right) - 1} \right] \times \frac{1}{\sqrt 2 - 1}$$

where $0\le EN\left({d}^{\left(k\right)}\right)\le 1$ and $p, q\epsilon [\mathrm{0,1}]$ are two fixed numbers. Then, the entropy weights of the $k$-th DM are determined as follows:

6 $$\lambda_{k} = \frac{{1 - EN\left( {d^{\left( k \right)} } \right)}}{{K - \mathop \sum \nolimits_{k = 1}^{K} EN\left( {d^{\left( k \right)} } \right)}}$$

where $${\lambda }_{k}\epsilon [\mathrm{0,1}], k=\mathrm{1,2},\dots K, {\sum }_{k=1}^{K}{\lambda }_{k}=1$$.

#### Obtaining the individual pairwise comparison matrices

Each DM is asked to provide his/her judgement regarding each dimension and the related indicators through individual pairwise comparison evaluation matrices. Linguistic terms, which are shown in Table 3, are used to evaluate the dimension and indicators. The collected data were transformed into IVIFNs, as shown in Table 3. Next, the corresponding individual pairwise comparison matrix for the $k$th DM used to evaluate the dimensions can be constructed as follows:

7 $$T_{iy}^{\left( k \right)} = \left[ {t_{iy}^{\left( k \right)} } \right]_{n \times n} = \left[ {\begin{array}{*{20}c} {\begin{array}{*{20}c} - \\ {t_{21} } \\ \end{array} } & {\begin{array}{*{20}c} {t_{12} } \\ - \\ \end{array} } & {\begin{array}{*{20}c} {\begin{array}{*{20}c} \ldots & {t_{1n} } \\ \end{array} } \\ {\begin{array}{*{20}c} \ldots & {t_{2n} } \\ \end{array} } \\ \end{array} } \\ \vdots & \vdots & {\begin{array}{*{20}c} { \vdots } & \vdots \\ \end{array} } \\ {t_{n1} } & {t_{n2} } & {\begin{array}{*{20}c} \ldots & - \\ \end{array} } \\ \end{array} } \right]^{\left( k \right)}$$

where $$t_{iy}^{\left( k \right)} = \left( {\left[ {\mu_{{t_{iy} }}^{L} , \mu_{{t_{iy} }}^{U} } \right], \left[ {\nu_{{t_{iy} }}^{L} , \nu_{{t_{iy} }}^{U} } \right], \left[ {\pi_{{t_{iy} }}^{L} , \pi_{{t_{iy} }}^{U} } \right]} \right)^{\left( k \right)}$$$$, i,y=\mathrm{1,2},\dots ,n;i\ne y$$, and $${{t}_{iy}}^{\left(k\right)}$$ denotes the evaluation importance degree of dimension $$i$$ over dimension $$y$$ according to DM$$k$$. In the same way, the individual pairwise comparison matrices for the $$j$$ th indicator with respect to the main $$i$$ th dimension can be obtained.

#### Calculating the aggregated IVIFNs and obtaining the group evaluation IVIFNs

Interval-valued intuitionistic fuzzy weighted averaging (IIFWA) is applied to aggregate the evaluation matrix ${{T}_{iy}}^{\left(k\right)}= {\left[{{t}_{iy}}^{\left(k\right)}\right]}_{n\times n}$ with ${T}^{k}= {\left[{{t}_{i}}^{\left(k\right)}\right]}_{n\times 1}$ for *k* = 1,…,*K*. The IIFWA operator inputs the vector of the weights of the DMs, as illustrated in formula (8).

$${{t}_{i}}^{\left(k\right)}=\mathrm{IVIFWA}\left({{t}_{i1}}^{\left(k\right)}, {{t}_{i2}}^{\left(k\right)},., {{t}_{in}}^{\left(k\right)}\right)=$$

8 $$\left( {\left[ {1 - \mathop \prod \limits_{y = 1}^{n} \left( {1 - \left( {\mu_{{t_{iy} }}^{L} } \right)^{\left( k \right)} } \right)^{{\lambda_{k} }} ,1 - \mathop \prod \limits_{y = 1}^{n} \left( {1 - \left( {\mu_{{t_{iy} }}^{U} } \right)^{\left( k \right)} } \right)^{{\lambda_{k} }} ,} \right], \left[ {\mathop \prod \limits_{y = 1}^{n} \left( {1 - \left( {\nu_{{t_{iy} }}^{L} } \right)^{\left( k \right)} } \right)^{{\lambda_{k} }} ,\mathop \prod \limits_{y = 1}^{n} \left( {1 - \left( {\nu_{{t_{iy} }}^{U} } \right)^{\left( k \right)} } \right)^{{\lambda_{k} }} } \right]} \right)$$

where ${{t}_{i}}^{\left(k\right)}$ represents the importance degree for the *i-*th dimension, where $i=1,\dots ,n$.

Additionally, for the *k*th DM, the IVIFN is given by $t_{i}^{\left( k \right)} = \left( {\left[ {\mu_{{t_{i} }}^{L} , \mu_{{t_{i} }}^{U} } \right], \left[ {\nu_{{t_{i} }}^{L} , \nu_{{t_{j} }}^{U} } \right], \left[ {\pi_{{t_{i} }}^{L} , \pi_{{t_{i} }}^{U} } \right]} \right)^{\left( k \right)}$. The IVIF values for the $j$th indicators with respect to their respective *i*-th dimension are computed in similar form. Finally, the IVIF values, which are based on the DMs’ evaluations, are aggregated into a unique main dimension by utilizing the IVIFWA operator, as shown in the following expression (Xu and Cai 2012):

9 $$t_{i} = {\text{IVIFWA}}\left( {t_{i}^{\left( 1 \right)} , t_{i}^{\left( 2 \right)} ,., t_{i}^{\left( K \right)} } \right) = \left( {\left[ {1 - \mathop \prod \limits_{k = 1}^{K} \left( {1 - \left( {\mu_{{t_{i} }}^{L} } \right)^{\left( k \right)} } \right)^{{\lambda_{k} }} ,1 - \mathop \prod \limits_{k = 1}^{K} \left( {1 - \left( {\mu_{{t_{i} }}^{U} } \right)^{\left( k \right)} } \right)^{{\lambda_{k} }} ,} \right], \left[ {\mathop \prod \limits_{k = 1}^{K} \left( {1 - \left( {\nu_{{t_{i} }}^{L} } \right)^{\left( k \right)} } \right)^{{\lambda_{k} }} ,\mathop \prod \limits_{k = 1}^{K} \left( {1 - \left( {\nu_{{t_{i} }}^{U} } \right)^{\left( k \right)} } \right)^{{\lambda_{k} }} } \right]} \right)$$

where ${\lambda }_{k}\epsilon [\mathrm{0,1}], {\sum }_{k=1}^{K}{\lambda }_{k}=1$ and ${t}_{i}$ indicates a group decision IVIF value for the *i*-th dimension, where *i* = 1,…,*n* and is represented as $t_{i} = \left( {\left[ {\mu_{{t_{i} }}^{L} , \mu_{{t_{i} }}^{U} } \right], \left[ {\nu_{{t_{i} }}^{L} , \nu_{{t_{i} }}^{U} } \right], \left[ {\pi_{{t_{i} }}^{L} , \pi_{{t_{i} }}^{U} } \right]} \right)$. The same procedure is applied to obtain group decision IVIFNs for the $j$ th indicator with respect to the *i*-th dimension.

#### Obtaining consistency ratio (CR)

The CR is useful for evaluating the extent to which each DM assessment is reliable and comprises acceptable measures of variation. Using the dimensions and their indicators as inputs, the CR is calculated. The random index (RI), for which the respective values are provided in Table 4, is applied here to compute the CR. The formula proposed by Saaty (1990) is utilized to calculate the CR, as shown below.

10 $$CR = \frac{{RI - \frac{{\sum \pi_{{t_{i} }}^{U} }}{n}}}{n - 1}$$

where *n* represents the number of dimensions and ${\pi }_{{t}_{i}}^{U}$ indicates the upper hesitancy value. The CRs for all indicators included in this analysis are calculated in similar form. According to Saaty (1990), a CR is considered acceptable if it has a value of 0.10 or lower (CR ≤ 0.10). If the CR is higher than 0.10 (CR > 0.10), the DM’s assessment is considered inconsistent and therefore should be either revised or dropped.

**Table 4:** Random indexes

*S*	1–2	3	4	5	6	7	8	9
*RI*	0.00	0.58	0.90	1.12	1.24	1.32	1.41	1.45

#### Calculating the weights of the dimensions and indicators

The weights of the dimensions and their indicators are objectively determined by using the IVIF values as inputs (see Table 3). The dimension weights, denoted by ${W}_{i}$, where ${W}_{i}\epsilon \left[\mathrm{0,1}\right]; i=\mathrm{1,2},\dots n; {\sum }_{i=1}^{n}{W}_{i}=1$, are obtained using the equations proposed by Büyüközkan et al. (2020), as illustrated in (11) and (12).

11 $$W_{i} = \frac{{1 - H\left( {c_{i} } \right)}}{{n - \mathop \sum \nolimits_{i = 1}^{n} H\left( {c_{i} } \right)}}$$

where

12 $$H\left( {c_{i} } \right) = 1 - \frac{{\mathop \sum \nolimits_{k = 1}^{K} \frac{{\lambda_{k} \left[ {\mu_{{t_{i} }}^{L} + \mu_{{t_{i} }}^{U} } \right]}}{2}}}{{\sqrt {\mathop \sum \nolimits_{k = 1}^{K} \frac{{\lambda_{k} \left[ {\left( {\mu_{{t_{i} }}^{L} } \right)^{2} + \left( {\mu_{{t_{i} }}^{U} } \right)^{2} + \left( {\nu_{{t_{i} }}^{L} } \right)^{2} + \left( {\nu_{{t_{i} }}^{U} } \right)^{2} } \right]}}{2}} }}$$

For each $i$th dimension ($i=\mathrm{1,2},\dots n$), the local weights for indicators ${C}_{ij}$, denoted by ${w}_{j}$_,_ where (${w}_{j}\epsilon \left[\mathrm{0,1}\right];\, j=\mathrm{1,2},\dots m; {\sum }_{j=1}^{n}{w}_{j}=1)$, are obtained by recursively utilizing formulas (11) and (12) for each dimension.

#### Ranking the dimensions and indicators

This last step comprises three types of results. First, the overall relative importance of each dimension (denoted by ${W}_{i}$) is calculated. The local weight for each indicator allocated to the *i*-th dimension is calculated (identified as ${w}_{j}$). The global weights that represent the relative importance of all indicators with respect to the goal are then calculated.

The abovementioned IVIF-AHP framework is used for an original application of linguistic scales and three types of IFS, namely, membership, non-membership and hesitation, to rank the relevant COVID-19 post-lockdown indicators. By using this methodology, the comparison matrix judgements typical of AHP problems yield more accurate results, as shown in the following section.

## Results

It is relevant for governments and DMs to evaluate the importance of the set of indicators introduced in Table 1 when designing a post-COVID-19 lockdown reopening strategy. Based on data collected from a group of DMs, a practical case study is presented in this section. To ensure the explanation is “easy-to-follow”, this illustrative case study follows the same eight-step methodology presented in the last section.

### Defining the goal, dimensions and indicators

**Fig. 3:**
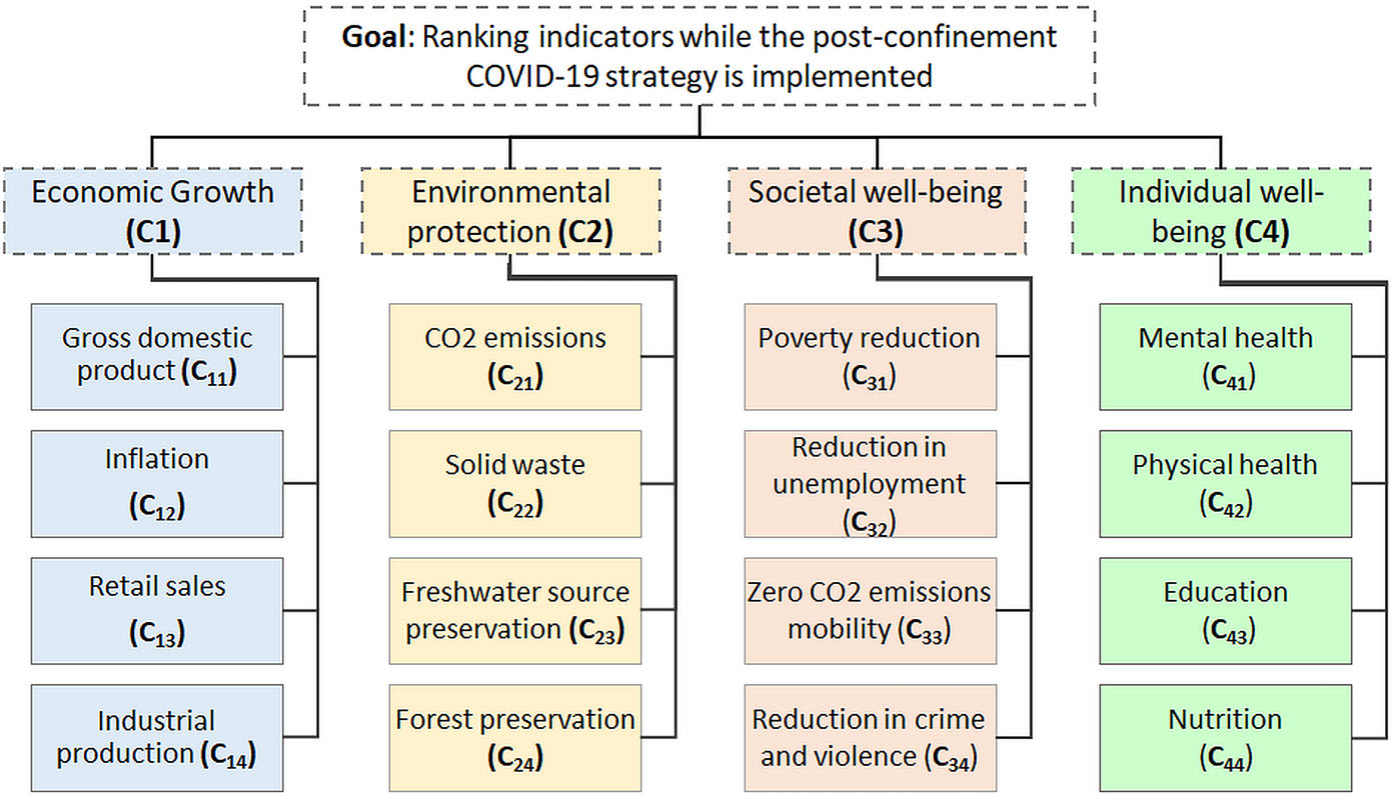
The hierarchical model of the indicators that should be considered for a post-COVID-19 lockdown reopening strategy

The structure of the IVIF-AHP problem consists of four main dimensions: economic growth, environmental protection, societal well-being and individual well-being. Similarly, there are 16 indicators that are hierarchically allocated to these dimensions. The overall framework is presented in Fig. 3. The operational definitions of the indicators, which includes a detailed explanation of each concept based on a review of the literature, is provided in Appendix 1. The next step consists of determining the group of experts that will participate in the study.

### Determining the group of DMs

A committee consisting of five experts,${e}_{k} (k=\mathrm{1,2},\dots 5)$ who were knowledgeable in areas such as the decision sciences, business, engineering and computer sciences was invited to participate. The group included professionals from both academia and industry. All participants were highly experienced in assessing indicators and working with multi-criteria decision analysis problems. The participants were asked to provide their assessment by responding to an electronic questionnaire that was designed for these purposes and featured the typical hierarchical structure of AHP problems. The academic background and profile of each participant is presented in Table 5.

**Table 5:** Academic backgrounds and profiles of the DMs

DM	Academic background	Profile
1	PhD in Computer Science	Head of the research centre at a financial institution
2	PhD in Business and Marketing	Professor at a private university
3	PhD in Systems Engineering	Professor and Chair of Decision and System Sciences Centre at a public university
4	PhD in Decision Science	Associate Professor and Head of the Quantitative Methods Department at a public university
5	PhD in Statistics and Data Science	Professor and Director of the Statistical Consulting Centre at a public university

### Computing the weights of the DMs

Considering the linguistic terms and their respective IVIFNs (see Table 1), the weights of the DMs ${e}_{k} (k=\mathrm{1,2},\dots 5)$ were calculated. First, the DMs were asked to evaluate their knowledge of the indicators that might affect reopening strategies following COVID-19 lockdowns. Later, the scale presented in Table 2 was used for these purposes and adapted to our electronic questionnaire. The weight of each DM in terms of the linguistic terms and IVIFN are presented in Table 6. Note that formulas (5) and (6) were applied to calculate these weights.

**Table 6:** Importance ratings of the DMs in terms of the linguistics terms and related weights

DM	Linguistic term	IVIFNs	Weights ($\lambda )$
DM1	Very knowledgeable (VK)	([0.80,0.85],[0.05,0.10],[0.05,0.15])	0.20
DM2	Moderately knowledgeable (MK)	([0.60,0.65],[0.10,0.15],[0.20,0.30])	0.19
DM3	Moderately knowledgeable (MK)	([0.60,0.65],[0.10,0.15],[0.20,0.30])	0.19
DM4	Extremely knowledgeable (EK)	([0.95,1.00],[0.00,0.00],[0.00,0.05])	0.22
DM5	Very knowledgeable (VK)	([0.80,0.85],[0.05,0.10],[0.05,0.15])	0.20

### Obtaining the individual pairwise comparison matrices

By using the linguistic terms shown in Table 3, DMs assessed the importance of each dimension with respect to the others and then the relative importance of indicators. The responses provided by the DMs regarding each comparison is provided in Appendix 2. The linguistic terms reported by the DMs were later transformed to IVIFNs by following the equivalences shown in Table 3. Note that formula (7) was utilized to generate the pairwise comparison matrices. A total of five matrices were computed (see Appendix 2). While the first matrix referred to the dimensions, the remaining four matrices related the same number of indicator groups.

### Calculating the aggregated IVIFNs and obtaining the group evaluation IVIFNs

By employing the IVIFNs, the equivalences, dimensions and indicators are aggregated with DMs’ respective weights through the application of formula (8). The output of this process in terms of the dimensions is provided in Table 7. Table 7 provides only the aggregated IVIFNs of the main dimensions. Later, the same calculation is replicated to aggregate the indicator evaluations into their respective dimensions by considering the IVIFN equivalences.

**Table 7:** Aggregated IVIFNs for the main dimensions

DMs	Dimensions	IVIFNs
DM_1_	C_1_	$([0.11, 0.45], [0.15, 0.55], [\mathrm{0.00,0.75}])$
	C_2_	$([\mathrm{0.14,0.31}], [0.17, 0.69], [\mathrm{0.00,0.69}])$
	C_3_	$([0.34, 0.52], [0.13, 0.48], [\mathrm{0.00,0.53}])$
	C_4_	$([0.13, 0.43], [0.14, 0.57], [\mathrm{0.00,0.74}])$
DM_2_	C_1_	$([0.13, 0.40], [0.15, 0.60],[0.00, 0.72])$
	C_2_	$([0.12, 0.37], [0.23, 0.63], [0.00, 0.64])$
	C_3_	$([0.15, 0.37], [0.12, 0.63], [0.00, 0.74])$
	C_4_	$([0.12, 0.43], [0.19, 0.57], [\mathrm{0.00,0.69}])$
DM_3_	C_1_	$([0.15, 0.39], [0.14, 0.61], [0.00, 0.71])$
	C_2_	$([0.12, 0.36], [0.13, 0.64], [0.00, 0.74])$
	C_3_	$([0.11, 0.40], [0.17, 0.60], [0.00, 0.72])$
	C_4_	$([0.10, 0.42], [0.13, 0.58], [0.00, 0.77])$
DM_4_	C_1_	$([0.18, 0.46], [\mathrm{0.09,0.54}], [0.00, 0.73])$
	C_2_	$([0.15, 0.44], [0.09, 0.56], [\mathrm{0.00,0.76}])$
	C_3_	$([0.18, 0.44], [0.13, 0.55], [0.00, 0.70])$
	C_4_	$([0.15, 0.43], [0.27, 0.57], [0.00, 0.57])$
DM_5_	C_1_	$([0.15, 0.42], [0.13, 0.58], [0.00, 0.73])$
	C_2_	$([0.26, 0.36], [0.27, 0.64], [0.00, 0.47])$
	C_3_	$([0.22, 0.42], [0.22, 0.58], [0.00, 0.56])$
	C_4_	$([0.23, 0.40], [0.30, 0.60], [0.00, 0.48])$

The IVIFWA operator, previously introduced in formula (9), is used here. Table 8 illustrates the outputs regarding the dimensions and four groups of indicators.

**Table 8:** Group evaluation of the IVIFNs for the main dimensions and the corresponding indicators

*IVIFNs for the main dimensions*	
C_1_	$([0.15, 0.43], [0.13, 0.57], [0.00, 0.73])$
C_2_	$([0.16, 0.37], [0.16, 0.63], [0.00, 0.68])$
C_3_	$([0.20, 0.43], [0.15, 0.57], [0.00, 0.65])$
C_4_	$([0.15, 0.42], [0.19, 0.58], [0.00, 0.66])$
*IVIFNs for the indicators of economic growth (C*_*1*_*)*	
C_11_	$([0.17, 0.38], [0.13, 0.62], [0.00, 0.71])$
C_12_	$([0.14, 0.41], [0.13, 0.58], [0.00, 0.73])$
C_13_	$([0.16, 0.42], [0.19, 0.57], [0.00, 0.66])$
C_14_	$([0.14, 0.43], [0.16, 0.57], [0.00, 0.70])$
*IVIFNs for the indicators of environmental protection (C*_*2*_*)*	
C_21_	$([0.17, 0.43], [0.21, 0.57], [0.00, 0.62])$
C_22_	$([0.14, 0.44], [0.22, 0.56], [0.00, 0.64])$
C_23_	$([0.21, 0.40], [0.17, 0.60], [0.00, 0.62])$
C_24_	$([0.23, 0.38], [0.19, 0.62], [0.00, 0.58])$
*IVIFNs for the indicators of societal well-being (C*_*3*_*)*	
C_31_	$([0.15, 0.38], [0.16, 0.62], [0.00, 0.69])$
C_32_	$([0.21, 0.42], [0.16, 0.58], [0.00, 0.62])$
C_33_	$([0.13, 0.39], [0.22, 0.61], [0.00, 0.65])$
C_34_	$([0.28, 0.50], [0.19, 0.50], [0.00, 0.53])$
*IVIFNs for the indicators of individual well-being group (C*_*4*_*)*	
C_41_	$([0.15, 0.42], [0.16, 0.58], [0.00, 0.68])$
C_42_	$([0.14, 0.43], [0.21, 0.57], [0.00, 0.65])$
C_43_	$([0.17, 0.36], [0.13, 0.55], [0.09, 0.71])$
C_44_	$([0.16, 0.39], [0.17, 0.52], [0.09, 0.67])$

### Obtaining the CR

The CRs for the dimensions group evaluation IVIFNs were calculated. By using formula (10), the CR for the dimensions can be obtained as follows.

$$CR = \frac{{0.90 - \frac{{\left( {0.73 + 0.68 + 0.65 + 0.66} \right)}}{4}}}{3} = 0.074$$

Similarly, the CRs were obtained for all IVIFNs; the CRs were 0.067, 0.095, 0.092 and 0.074 for C_1_, C_2_, C_3_ and C_4_, respectively. Note that all the CR values were below 0.10; therefore, all aggregation matrices are consistent.

### Calculating the weights for the dimensions and associated indicators

By applying formulas (11) and (12), the weights for the dimensions and indicators are obtained. Local weights, which refer to the relative importance to indicators within respect to its groups, are calculated in similar form. Based on the above, three types of weights are provided. The first weight is related to dimension importance, followed by local and global indicator weights. The weight values of all dimensions and the local weight values of the indicators included in the study are shown in Table 9. The global weights are provided in Table 10.

**Table 9:** Weights of the main dimensions and associated indicators

Dimension	Weights ($W$)	Indicators	Local weights ($w$)
Societal well-being (C_3_)	0.2722	Reduction in crime and violence (C_34_)	0.2905
		Reduction in unemployment (C_32_)	0.2680
		Poverty reduction (C_31_)	0.2243
		Zero CO2 emissions mobility (C_33_)	0.2172
Economic growth (C_1_)	0.2541	Retail sales (C_13_)	0.2574
		Industrial production (C_14_)	0.2527
		Inflation (C_12_)	0.2511
		Gross domestic product (C_11_)	0.2388
Individual well-being (C_4_)	0.2472	Nutrition (C_44_)	0.2528
		Mental health (C_41_)	0.2519
		Physical health (C_42_)	0.2513
		Education (C_43_)	0.2440
Environmental protection (C_2_)	0.2265	Freshwater source preservation (C_23_)	0.2535
		CO2 emissions (C_21_)	0.2520
		Forest preservation (C_24_)	0.2489
		Solid waste (C_22_)	0.2456

**Table 10:** Global weights of the indicators

Indicator	Global weight
Reduction in crime and violence (C_34_)	0.072625
Reduction in unemployment (C_32_)	0.067000
Retail sales (C_13_)	0.064350
Freshwater source preservation (C_23_)	0.063375
Nutrition (C_44_)	0.063200
Industrial production (C_14_)	0.063175
CO2 emissions (C_21_)	0.063000
Mental health (C_41_)	0.062975
Physical health (C_42_)	0.062825
Inflation (C_12_)	0.062775
Forest preservation (C_24_)	0.062225
Solid waste (C_22_)	0.061400
Education (C_43_)	0.061000
Gross domestic product (C_11_)	0.059700
Poverty reduction (C_31_)	0.056075
Zero CO2 emissions mobility (C_33_)	0.054300

### Ranking the dimensions and indicators

The ranking of the dimensions, based on their weights, is carried out. “Societal well-being” (C_3_) is the most important dimension, followed by “Economic growth” (C_1_), “Individual well-being” (C_4_) and “Environmental protection” (C_2_).

In relation to the global weights, the five most important indicators are “Reduction in crime and violence” (C_32_), “Reduction in unemployment” (C_32_), “Retail sales*”* (C_13_), “Freshwater source preservation” (C_23_) and “Nutrition” (C_23_). Note that “Societal well-being” and “Environmental protection” are the two dimensions with four indicators. The complete ranking of all indicators with respect to the global weights is provided in Table 10.

## Discussion

A DSS is a tool for collecting, organizing and analysing information to solve decision-making problems. A DSS can be used for either single or GDM by ensuring the results are more robust when dealing with complex or unstructured scenarios. In this paper, a DSS is proposed to help stakeholders by prioritizing indicators for designing and implementing a post-COVID-19 strategy. Considering that COVID-19 pandemic and related lockdowns are complex scenarios, mainly featuring vagueness and uncertainty, the proposed tool is based on the IFS AHP (Abdullah and Najib 2016). By distinguishing among membership, non-membership and hesitancy, our DSS is capable of more accurately calculating the DM’s assessments. Four dimensions are considered to shape the main framework of the problem. International organizations, such as the United Nations, World Bank and International Labour Organization, among others, widely utilize these dimensions-indicators for ranking, comparing or investigating countries (FAO 2021b; ILO 2021; IMF 2021; OECD 2021a; UN 2021; WHO 2021). Each dimension comprises four indicators. Our DSS was utilized by DMs from industry and academia. The assessment made by the group of DMs yielded three types of results: dimension importance and the prioritization of the global and local indicators.

“Societal well-being” was the most important dimension, followed by “Economic growth”, “Individual well-being” and “Environmental protection”. Addressing poverty by guaranteeing access to material resources to help individuals achieve acceptable levels of quality of life and ensuring that worthy sources of employment are available in a clean and safe environment, should be key aspects of post-COVID-19 strategies. According to Bick and Blandin (2020), the unemployment rate in the U.S. increased to 20% by the beginning of May 2020. Martin et al. (2020) state that unemployment is among the most devastating consequences of policies that restrict movement and lockdowns, since unemployed individuals are mainly focused on meeting their primary needs (e.g., food, housing and clothing), and therefore, the risk of being infected is secondary. Successful COVID-19 prevention policies are inversely related to unemployment. Ensuring socioeconomic stability and employment confidence after lockdowns must be a priority for governments when post-COVID lockdown policies are designed and implemented (Tran et al. 2020).

Research provides evidence of the negative effects of lockdowns on individuals’ mental health. The negative effects are manifested as anxiety, depression or eating disorders that eventually lead to several types of violence (Piquero et al. 2020). On the other hand, crime and violence events are products of these circumstances. The likelihood that crime or violence occurs is given by the convergence of the perpetrator and victim in the absence of surveillance (Estévez-Soto 2020). Therefore, lockdowns lead to a decrease in robberies in public places but an increase in several types of domestic violence, since the perpetrators and victims spend more time at home. A study reported that from March to May 2020, there was a substantial decrease in robberies and residential burglaries but a significant increase in domestic violence in Los Angeles and Indianapolis (Mohler et al. 2020). Another study in Mexico City reported a significant increase in the number of calls received by the Violence Against Women (VAW) help line due to the lockdown. This increase is positively correlated with the number of domestic violence events indicated by official statistics (Estévez-Soto 2020).

As reported in the literature, crime, violence and unemployment are among the variables most negatively affected by lockdowns (Mohler et al. 2020). Our proposed DSS is consistent with this evidence, in the sense that governments and policy makers should consider these variables as priorities when the lockdowns are lifted. Note that the dimension “Environmental protection” appears last in terms of importance. This does not mean that protecting the environment is not relevant or unimportant, quite the opposite. Based on a general framework composed of conflicting and negatively correlated indicators, the DSS helps DMs focus their attention on the indicators that should be prioritized when the lockdowns are lifted. By differentiating between immediate priorities (Societal well-being) and long-term priorities (Environmental protection), the system helps policy makers by strategically focusing their attention on priorities, given the complex and unstructured situation.

## Conclusions

By the end of January 2021, the number of positive COVID cases worldwide was around 108, 148,755 and 2,267,768 deaths (JHU 2021). Humankind is facing the one of the biggest economic, social and public health crises. Besides the irreparable deaths and the related suffering to relatives, the pandemic is bringing devastating effects on income and employment of millions of families around the world, and therefore deteriorating the well-being on individuals and societies. As commonly occurs on this type of phenomena, the poorest part of the population is the most affected since lockdowns and curfews lead to higher levels of inequality, poverty and setbacks on nutrition, well-being and economic development. Since the emergence of the virus until January 2021, several countries passed from believing the pandemic was under control to face new lockdowns, which forced governments and policy makers to rethink strategies and actions. Although, plenty of scientific evidence related to key aspects of the pandemic emerged on the past few months, at this moment it is not possible to carry out an accurate assessment of how effective governments' strategies and actions have been, since the pandemic is not over.

Under this perspective, this study makes valuable contribution by making available to governments, policy makers and stakeholders a flexible and easily adaptable methodology, which can be replicated to assess future stages of the pandemic. Since the proposed framework is based on indicators introduced by organizations as World Health Organization (WHO), International Monetary Found (IMF) and Food and Agriculture Organization (FAO), stakeholders can find here a scientific-based approach for conducting further research related with the pandemic evolution.

In this work, we do not attempt to set the foundations for designing post-COVID lockdown strategies. Our goal is much more modest. Considering that the COVID pandemic is a phenomenon with multiple variables, we contribute to the discussion of how this complex problem should be approached and highlight additional elements for analyses. Given that the DMs participating in this study are not directly involved in public policy making, our results are more illustrative than prescriptive. Considering this framework is based on an approach that takes generic indicators proposed by international organizations, country level implementations should be carefully studied, since each country has peculiarities and not all proposed indicators might be suitable for all countries. Thus, we attempt to put at the hand of governments, policymakers and stakeholders, consistent and evidence-based tools to evaluate difficult choices and avoid further deterioration in the economy, employment and wellbeing for societies and individuals. Additionally, the present work illustrates the suitability of decision science tools for tackling complex and unstructured problems, such as the COVID pandemic, and consequently make an original contribution in that direction. Additional research involving expert public policy makers in the DSS process is needed to produce helpful results for society. Further research related to the present pandemic or other pandemics that might arise in the future can find in this work a set of general guidelines through which decision-making processes can be conducted within a clear and structured framework.

## Figures and Tables

**Table 11:**
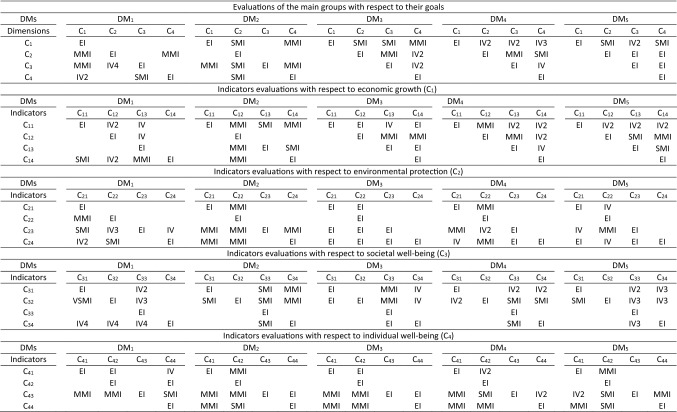
GDMs’ Judgement of dimensions and indicators by linguistic terms

## Data Availability

Data is available in “Appendix 2”.

## Appendix 1: Indicators definitions

### Economic growth

*Gross domestic product (GDP)* It is a well-known measure used to estimate the market value of all goods and services that are produced during a specific period of time. The Organization for Economic Co-operation and Development (OECD) defines the GDP as a measure of production that is calculated as follows: the sum of the gross value added to a country or region, plus taxes and minus any subsidies (OECDb 2020). Similarly, the International Monetary Fund (IMF) states that the GDP represents the monetary value of all final goods and services that are bought by final users and produced in a given country or region over a specific period of time (IMF 2020). Clearly, any negative effects on the GDP caused by COVID-19 lockdowns should be minimized. Reopening strategies should focus on facilitating the growth of this indicator.

*Inflation* It is a concept widely used in economics that denotes a continued increase in the general level of prices over a period of time (Işığıçok et al. 2020). One unit of a currency buys fewer goods and services every time there is an increase in the general price levels. Inflation represents a reduction in the purchasing power in an economy. Deflation is the opposite effect, a sustained decrease in the general price level. When designing reopening strategies after COVID-19 lockdowns, policymakers should consider keeping inflation as low as possible.

*Retail sales* Comprise all purchases of finished goods and services by either final consumers or businesses. Note that retail sales represent the middle link in the supply chain (Amadeo 2020). Manufacturers sell finished goods and services to retailers, who sell them to consumers. This indicator should be considered when designing reopening strategies since retail sales represent approximately two-thirds of the GDP.

*Industrial production* Represents all outputs of the industrial sector over a specific period of time. Although differences exist across countries, industrial production typically includes the production of three main industries: manufacturing, mining and utilities. Although the contribution of industrial production is relatively small with respect to other indicators, it should be considered since it is highly sensitive to interest rates, consumer demand and abrupt changes in consumption patterns (Shapiro et al. 1989; Hudson 2020). Therefore, it is important to consider this measure when developing a reopening strategy.

### Environmental protection

*CO2 emissions* CO2 is a colourless, odourless and non-poisonous gas formed by the combustion of carbon. Greenhouse gas emissions represent the release of CO2 to the atmosphere over a certain period of time (US-EIAa 2020). Over the last decade, evidence has shown that greenhouse gas emissions are among the main causes of global warming worldwide. According to Eurostat, the official website that provides statistics about the European Union, between 2008 and 2018, the level of CO2 emissions mainly due to the supply of electricity, gas, steam and air conditioning fell by 271 million tons of CO2 equivalents, a decrease of 22.2% in relative terms (Eurostat 2020).

*Solid waste* The Environmental Protection Agency (EPA) of the U.S. defines “solid waste” as refuse; mud from wastewater treatment plants, water supply treatment plants, and air pollution control stations; and other castoff materials. Solid waste is mainly produced by industrial, commercial, mining, and agricultural operations and individual activities. According to the EPA, nearly everything we do leaves behind can be considered “solid waste”. Note that the previous definition is not limited to garbage that is physically solid. Many types of solid wastes are liquid, colloidal suspensions or contain gaseous material (US-EIAb 2020; US-EPA 2020). One negative effect of the COVID-19 pandemic is the increasing demand for plastics to mitigate the spread of the disease, which has created a massive solid waste disposal problem. Current waste management systems worldwide are already unable to cope with solid waste at the rate that healthcare systems are increasingly demanding devices such as acrylic masks, mouth covers and plastic fences.

*Freshwater source preservation* Any naturally existing water except that flowing from seas and oceans is defined as fresh water. Fresh water includes water located at the ice caps, glaciers, icebergs, bogs, ponds, lakes, rivers and underground streams. One of the main characteristics of fresh water is low concentrations of dissolved salts, minerals and other solids (Beatley 2000). Note that the definition of fresh water does not include drinkable water. Most of the fresh water existing on the planet is unsuitable for drinking, and some type of treatment is needed. Due to the emergence of the novel COVID-219, a better understanding of how freshwater is used in large metropolitan centres is needed. When designing reopening strategies after COVID-19 lockdowns, policy makers should consider actions that will save and restore ecosystems, including freshwater sources (Beatley 2000).

*Forest preservation* According to the FAOa (2020), a forest is land that comprises more than 0.5 hectares with trees higher than 5 m and a canopy cover of more than 10%. In addition, trees and foliage are able to reach this height. Note that this definition does not include land reserved for agriculture or households (FAOa 2020). To maintain biodiversity, it is mandatory to preserve the structural complexity of forests. Understanding how pollution and other external agents affect the forest structure is crucial for the successful management of forest ecosystems (Collins et al. 2012).

*Poverty reduction* Traditionally, poverty is defined as the condition in which individuals lack the financial resources to achieve a minimum standard of living. Poverty refers to the inability to access opportunities and choices and is therefore a violation of human dignity (Barahona 2018). Poverty is operationalized as not having enough food or clothing or access to essential services such as potable water, education, and electricity for health services. Recently, the idea that poverty is a multidimensional concept rather than being limited to low-income status has gained importance. A comprehensive definition of poverty should include dimensions such as low self-esteem, poor physical conditions, insecurity, and illiteracy (Barahona 2018). Therefore, clearly, governments and policy makers should consider actions to mitigate poverty when developing reopening strategies after COVID-19 lockdowns.

*Reduction in unemployment* Generally, unemployment refers to the state of being without work while seeking employment. Economic and political sciences distinguish among several types of unemployment. Voluntary unemployment is attributed to an individual’s decision, and involuntary unemployment takes place mainly due to the socio-economic environment. According to the International Labour Organization, from April to June 2020, it is estimated that the unemployment rate increased from 5.4 to 14.0% worldwide (ILO 2020). Therefore, protecting jobs and implementing strategies to prevent increasing unemployment after COVID-19 lockdowns should be priorities for governments and policymakers.

*Zero CO2 emissions mobility* According to the European Commission, transport activities represent the main source of CO2 emissions overall and air pollution in large cities. There is evidence that emissions have been decreasing since 2007, although they are still higher than those in 1990. In 2014, mobility related to persons and goods represented approximately 70% of the total emissions in this sector. In April 2020, lockdowns and stay-at-home policies resulted in a 40% reduction in vehicle travel in the U.S.; it was estimated that CO2 emissions fell by 19% in comparison with the same period of the previous year (Cicala et al. 2020). The COVID-19 outbreak temporarily improved China’s air quality and significantly contributed to a reduction in global CO2 emissions. Governments should consider this trend when implementing new policies and take into account that clean air is essential for human beings**.**

*Reduction in crime and violence* Generally, violence is defined as “the use of physical force to injure, abuse, damage, or destroy”. The World Health Organization states that violence is “the intentional use of physical force or power, threatened or actual, against oneself, another person, or against a group or community, which either results in or has high likelihood of resulting in injury, death, psychological harm, maldevelopment, or deprivation” (WHO 2020). Crime and violence affect millions of individuals worldwide. Social and economic inequality are considered to be the main causes of an increase in violence. Impunity is another trigger for violence since justice systems fail to properly punish and prevent crime. Although crime and violence are related to several social and economic variables, recent studies have shown an inverse correlation between crime, violence and economic growth (Aboal et al. 2016). It is important that governments consider proper strategies to mitigate crime and violence as COVID-19 lockdowns are finishing and the “new normal” once lockdowns have been lifted.

*Mental health* According to the U.S. Department of Health and Human Services mental health “includes our emotional, psychological, and social well-being”. Mental health reflects an individual’s capability to handle stress, interact with others and make decisions (US–DHHS 2020). Recent studies found that anxiety and depression were the most frequent reactions to the COVID-19 pandemic. Lockdowns and social distancing have collateral effects such as stress, anxiety, depressive symptoms, insomnia and anger. Health organizations and medical centres should recommend strategies to mitigate these negative effects. Although it is known individuals have been affected by these symptoms due to COVID-19, more research is needed to better understand the long term effects of mental health issues.

*Physical health* According to WHO guidelines, the minimal amount of physical activity required for adults aged between 18 and 64 years is at least 150 min of moderate-intensity activity per week (Wu and McGoogan 2020). On the other hand, restrictions on physical activity (e.g., as a consequence of the lockdowns and quarantines) are associated with unfavourable metabolic effects that eventually might increase the risk of chronic diseases such as diabetes, cancer, osteoporosis or cardiovascular disease. The lack of physical activity also has negative consequences for mental health, which could lead to anger, anxiety, frustration or depression (Brooks et al. 2020). Several studies suggest staying active during and after lockdowns, since physical activity is essential for mental and physical health. Physical activity can also prevent COVID-19 from evolving into a severe case.

*Education* It is defined as the process of facilitating learning or the acquisition of knowledge, skills, values, beliefs, and habits. Education can be delivered or transferred through teaching, training, storytelling or direct research. Formal education can be distinguished from informal education. The former is based on structured methodologies and environments, and usually, its main purpose is teaching students. On the other hand, informal education is mainly obtained through life experiences occurring outside the classroom. Informal education takes place through conversations, experiences and engagement in various situations (Kjällander et al. 2018). Education (formal or informal) is of strategic importance when combating COVID-19, since educated individuals are more capable of reducing the spread of new infections. Education plays a key role in mitigating the spread of COVID-19. Therefore, governments should consider this indicator at the moment reopening strategies are implemented.

*Nutrition* is defined by the US National Library of Medicine as the use of food and other nourishing materials by the body. This process comprises three steps. The first takes place when food is eaten or drunk. Second, the body breaks down what has been consumed into nutrients. Finally, nutrients travel through the bloodstream to different organs, which transforms them into “energy”. According to the U.S. National Cancer Institute, nutrition science is multidisciplinary and important for biomedicine, agriculture, public policy and health promotion, among others. Individuals with access to balanced diets are able to strengthen their immune system and therefore are less likely to become sick. Balanced nutrition contributes to reducing infections and the progression of COVID-19 and reducing the recovery time (Jaggers et al. 2020). Policy makers and governments should understand the importance of nutrition in the context of the COVID-19 pandemic and the “new normal”, since adequate nutrition leads to fewer deaths.

## Appendix 2

See Table11.

## Code availability

No applicable.
