# Correction to: Decision support system for ranking relevant indicators for reopening strategies following COVID-19 lockdowns

**DOI:** 10.1007/s11135-021-01154-2

**Published:** 2021-05-03

**Authors:** Tarifa S. Almulhim, Igor Barahona

**Affiliations:** 1 Department of Quantitative Methods, School of Business, King Faisal University, P.O.Box 400, Al-Ahsa, 31982 Saudi Arabia; 2 Laboratory of Applications of Mathematics, Institute of Mathematics, Universidad Nacional Autónoma de México (UNAM), 62243 Cuernavaca City, Morelos México

## Correction to: Quality & Quantity 10.1007/s11135-021-01129-3

Unfortunately, the acknowledgment text was incompletely published in the original article. The complete acknowledgment text is provided in this correction.

